# 9-(3,4-Dimeth­oxy­phen­yl)-3,4,5,6,7,9-hexa­hydroxanthene-1,8(2*H*)-dione

**DOI:** 10.1107/S1600536811017867

**Published:** 2011-05-20

**Authors:** Sayed Hasan Mehdi, Rokiah Hashim, Raza Murad Ghalib, Chin Sing Yeap, Hoong-Kun Fun

**Affiliations:** a School of Industrial Technology, Universiti Sains Malaysia, 11800 USM, Penang, Malaysia; b X-ray Crystallography Unit, School of Physics, Universiti Sains Malaysia, 11800 USM, Penang, Malaysia

## Abstract

In the title compound, C_21_H_22_O_5_, the mean planes of the pyran and dimeth­oxy­phenyl rings are nearly perpendicular to one another, with the dihedral angle between them being 88.21 (8)°. The pyran ring adopts a boat conformation whereas the two fused cyclo­hexane rings adopt envelope conformations. In the crystal, mol­ecules are linked into a three-dimensional network by inter­molecular C—H⋯O hydrogen bonds.

## Related literature

For condensation reactions between carbonyl compounds with active methyl­ene compounds, see: Chalais *et al.* (1985 ▶); Prajapati & Sanduh (1993 ▶); Texier-Boullet & Foucaud (1982 ▶); Jone (1967 ▶). For the stability of the temperature controller used in the data collection, see: Cosier & Glazer (1986 ▶). For ring conformations, see: Cremer & Pople (1975 ▶).
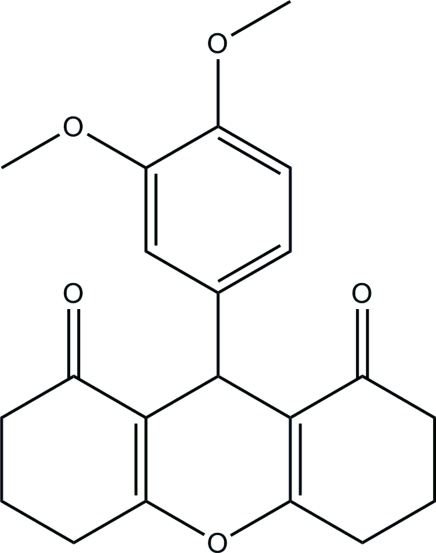


## Experimental

### 

#### Crystal data

C_21_H_22_O_5_
*M* *_r_* = 354.39
Monoclinic, 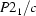

*a* = 8.7733 (3) Å
*b* = 15.2246 (5) Å
*c* = 14.6646 (4) Å
β = 116.891 (2)°
*V* = 1746.95 (10) Å^3^
*Z* = 4
Mo *K*α radiation
μ = 0.10 mm^−1^
*T* = 100 K
0.58 × 0.30 × 0.18 mm

#### Data collection

Bruker SMART APEXII CCD area-detector diffractometer
Absorption correction: multi-scan (*SADABS*; Bruker, 2009 ▶) *T*_min_ = 0.947, *T*_max_ = 0.983
19254 measured reflections
5118 independent reflections
4066 reflections with *I* > 2σ(*I*)
*R*_int_ = 0.042

#### Refinement

*R*[*F* ^2^ > 2σ(*F* ^2^)] = 0.048
*wR*(*F* ^2^) = 0.130
*S* = 1.05
5118 reflections
237 parameters
H-atom parameters constrained
Δρ_max_ = 0.35 e Å^−3^
Δρ_min_ = −0.28 e Å^−3^

### 

Data collection: *APEX2* (Bruker, 2009 ▶); cell refinement: *SAINT* (Bruker, 2009 ▶); data reduction: *SAINT*; program(s) used to solve structure: *SHELXTL* (Sheldrick, 2008 ▶); program(s) used to refine structure: *SHELXTL*; molecular graphics: *SHELXTL*; software used to prepare material for publication: *SHELXTL* and *PLATON* (Spek, 2009 ▶).

## Supplementary Material

Crystal structure: contains datablocks global, I. DOI: 10.1107/S1600536811017867/bq2301sup1.cif

Structure factors: contains datablocks I. DOI: 10.1107/S1600536811017867/bq2301Isup2.hkl

Supplementary material file. DOI: 10.1107/S1600536811017867/bq2301Isup3.cml

Additional supplementary materials:  crystallographic information; 3D view; checkCIF report

## Figures and Tables

**Table 1:** Hydrogen-bond geometry (Å, °)

*D*—H⋯*A*	*D*—H	H⋯*A*	*D*⋯*A*	*D*—H⋯*A*
C3—H3*A*⋯O4^i^	0.99	2.41	3.3647 (18)	161
C5—H5*A*⋯O5^ii^	0.99	2.56	3.2366 (17)	126
C9—H9*A*⋯O2^ii^	0.99	2.55	3.3210 (17)	135
C10—H10*B*⋯O5^iii^	0.99	2.50	3.4822 (19)	173
C21—H21*C*⋯O2^iv^	0.98	2.57	3.1069 (18)	114

Symmetry codes: (i) 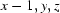; (ii) 
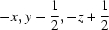
; (iii) 
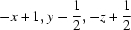
; (iv) 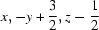.

**Fig. 1.**
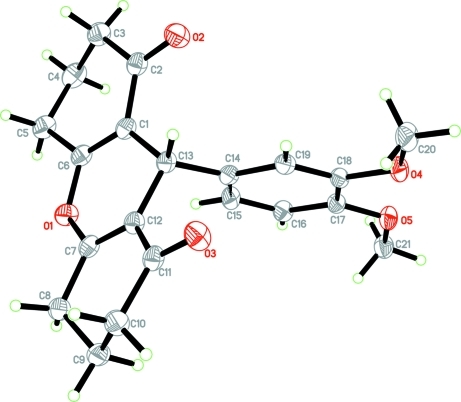
The molecular structure of the title compound, with atom labels and 50% probability ellipsoids for non-H atoms.

**Fig. 2.**
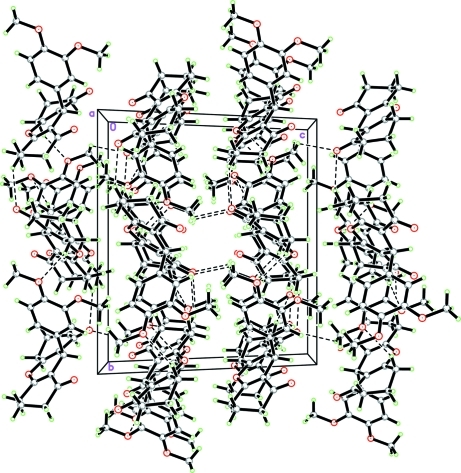
The crystal packing of title compound, viewed down a axis, showing molecules linked into a three-dimensional network. Hydrogen bonds are shown as dashed lines.

## supplementary crystallographic information

### Comment

The most important synthetic method for the preparation of substituted alkenes is the condensation reaction between carbonyl compounds with active methylene compounds. Xonotlite, cadmium iodide aluminium oxide and other Lewis acids and bases have been previously used for such type of reactions (Chalais *et al.*, 1985; Prajapati & Sanduh, 1993; Texier-Boullet & Foucaud, 1982; Jone, 1967). In this paper we are reporting the synthesis of title compound (Fig. 1) by simple heating of 1,3-cyclohexanedione with veratraldehyde in acetic acid without the use of any catalyst. The structure is supported by spectral analysis like IR, ^1^H NMR, ^13^C NMR and finally confirmed by *x*-ray crystallography.

In the title compound, the mean plane of pyran ring and the dimethoxyphenyl ring are nearly perpendicular to each other with the dihedral angle between them being 88.21 (8)°. The dimethoxyphenyl ring is planar with the torsion angle of C20–O4–C18–C19 = -5.34 (18)° and C21–O5–C17–C16 = -5.67 (18)°. The two cyclohexane rings adopt envelope conformations [puckering amplitude Q = 0.4284 (16) Å, θ = 124.3 (2)°, φ = 359.0 (3)°; Q = 0.4899 (15) Å, θ = 57.91 (18)°, φ = 132.8 (2)°, whereas the pyran ring adopt a boat conformation [Q = 0.2125 (13) Å, θ = 77.9 (4)°, φ = 187.0 (4)°] (Cremer & Pople, 1975). In the crystal structure, the molecules are linked into a three-dimensional network (Fig. 2) by intermolecular C3—H3A···O4, C5—H5A···O5, C9—H9A···O2, C10—H10B···O5 and C21—H21C···O2 hydrogen bonds (Table 1).

### Experimental

A mixture of 1,3-cyclohexanedione (1.12 g m, 10 mmol) and veratraldehyde (1.66 g m, 10 mmol) was heated in 25 ml of glacial acetic acid for three hours. Completion of the reaction was monitored by TLC. The reaction mixture was dried on rotary evaporator under reduced pressure. The crude mixture thus obtained was successively treated with chloroform and ethanol. The ethanol fraction on crystallization furnished cream colored crystals of title compound (yield 90%, m. pt. 216°C). IR (KBr) ν_max_: 2966, 2933, 2868, 2833, 1664, 1620, 1513, 1441, 1358, 1261, 1240, 1171, 1132, 1025, 956, 906, 859, 807, 726 cm^-1. 1^H NMR (300 MHz, DMSO-d_6_): δ1.75–1.88 (m, 2H), 1.94–2.06 (m, 2H), 2.20–2.38 (m, 42H), 2.48–2.68 (m, 4H), 4.21 (s, 1H), 6.78–6.96 (3*H*, m). ^13^C NMR (75 MHz, DMSO-d_6_): δ 21.6, 26.9, 30.2, 36.6, 56.8, 112.6, 114.4, 115.8, 123.2, 136.4, 146.2, 148.4, 198.2. IR spectrum was taken on Shimadzu IR-408 Perkin Elmer 1800 (FTIR). ^1^H NMR was recorded on Bruker Avance 300 MHz with TMS as an internal standard and 75 MHz for ^13^C NMR. Spectrum was recorded in DMSO-d_6_. The melting point was taken on Thermo Fisher digital melting point apparatus of IA9000 series and is uncorrected.

### Refinement

All hydrogen atoms were positioned geomatrically [C–H = 0.95–1.00 Å] and refined using a riding model, with *U*_iso_(H) = 1.2 or 1.5 *U*_eq_(C).

### Crystal data

**Table:**

C_21_H_22_O_5_	*F*(000) = 752
*M**_r_* = 354.39	*D*_x_ = 1.347 Mg m^−^^3^
Monoclinic, *P*2_1_/*c*	Mo *K*α radiation, λ = 0.71073 Å
Hall symbol: -P 2ybc	Cell parameters from 6186 reflections
*a* = 8.7733 (3) Å	θ = 2.6–30.0°
*b* = 15.2246 (5) Å	µ = 0.10 mm^−^^1^
*c* = 14.6646 (4) Å	*T* = 100 K
β = 116.891 (2)°	Block, yellow
*V* = 1746.95 (10) Å^3^	0.58 × 0.30 × 0.18 mm
*Z* = 4	

### Data collection

**Table:**

Bruker SMART APEXII CCD area-detector diffractometer	5118 independent reflections
Radiation source: fine-focus sealed tube	4066 reflections with *I* > 2σ(*I*)
graphite	*R*_int_ = 0.042
φ and ω scans	θ_max_ = 30.1°, θ_min_ = 2.1°
Absorption correction: multi-scan (*SADABS*; Bruker, 2009)	*h* = −12→9
*T*_min_ = 0.947, *T*_max_ = 0.983	*k* = −21→18
19254 measured reflections	*l* = −20→20

### Refinement

**Table:**

Refinement on *F*^2^	Primary atom site location: structure-invariant direct methods
Least-squares matrix: full	Secondary atom site location: difference Fourier map
*R*[*F*^2^ > 2σ(*F*^2^)] = 0.048	Hydrogen site location: inferred from neighbouring sites
*wR*(*F*^2^) = 0.130	H-atom parameters constrained
*S* = 1.05	*w* = 1/[σ^2^(*F*_o_^2^) + (0.0608*P*)^2^ + 0.471*P*] where *P* = (*F*_o_^2^ + 2*F*_c_^2^)/3
5118 reflections	(Δ/σ)_max_ < 0.001
237 parameters	Δρ_max_ = 0.35 e Å^−^^3^
0 restraints	Δρ_min_ = −0.28 e Å^−^^3^

### Special details

**Table:**

Experimental. The crystal was placed in the cold stream of an Oxford Cryosystems Cobra open-flow nitrogen cryostat (Cosier & Glazer, 1986) operating at 100.0 (1) K.
Geometry. All e.s.d.'s (except the e.s.d. in the dihedral angle between two l.s. planes) are estimated using the full covariance matrix. The cell e.s.d.'s are taken into account individually in the estimation of e.s.d.'s in distances, angles and torsion angles; correlations between e.s.d.'s in cell parameters are only used when they are defined by crystal symmetry. An approximate (isotropic) treatment of cell e.s.d.'s is used for estimating e.s.d.'s involving l.s. planes.
Refinement. Refinement of *F*^2^ against ALL reflections. The weighted *R*-factor *wR* and goodness of fit *S* are based on *F*^2^, conventional *R*-factors *R* are based on *F*, with *F* set to zero for negative *F*^2^. The threshold expression of *F*^2^ > σ(*F*^2^) is used only for calculating *R*-factors(gt) *etc*. and is not relevant to the choice of reflections for refinement. *R*-factors based on *F*^2^ are statistically about twice as large as those based on *F*, and *R*- factors based on ALL data will be even larger.

### Fractional atomic coordinates and isotropic or equivalent isotropic displacement parameters (Å^2^)

**Table:**

	*x*	*y*	*z*	*U*_iso_*/*U*_eq_	
O1	−0.23678 (10)	0.45054 (6)	0.11930 (7)	0.0253 (2)	
O2	−0.11008 (12)	0.63020 (7)	0.40709 (7)	0.0307 (2)	
O3	0.34649 (11)	0.44914 (7)	0.36290 (7)	0.0293 (2)	
O4	0.46323 (11)	0.77498 (6)	0.38751 (7)	0.0252 (2)	
O5	0.31222 (11)	0.84133 (6)	0.20530 (7)	0.0254 (2)	
C1	−0.16143 (15)	0.54404 (8)	0.26377 (9)	0.0212 (2)	
C2	−0.21561 (16)	0.59572 (9)	0.32848 (10)	0.0240 (3)	
C3	−0.40468 (17)	0.60323 (10)	0.29722 (11)	0.0296 (3)	
H3A	−0.4278	0.6619	0.3173	0.035*	
H3B	−0.4359	0.5588	0.3352	0.035*	
C4	−0.51736 (17)	0.59051 (10)	0.18363 (11)	0.0317 (3)	
H4A	−0.5069	0.6424	0.1461	0.038*	
H4B	−0.6381	0.5859	0.1706	0.038*	
C5	−0.46837 (16)	0.50797 (10)	0.14364 (10)	0.0269 (3)	
H5A	−0.5066	0.4553	0.1672	0.032*	
H5B	−0.5268	0.5083	0.0680	0.032*	
C6	−0.27955 (15)	0.50362 (9)	0.18052 (9)	0.0220 (2)	
C7	−0.06793 (15)	0.42479 (9)	0.15659 (9)	0.0219 (2)	
C8	−0.04677 (16)	0.35533 (9)	0.09158 (10)	0.0259 (3)	
H8A	−0.1171	0.3700	0.0185	0.031*	
H8B	−0.0871	0.2984	0.1051	0.031*	
C9	0.14076 (16)	0.34741 (9)	0.11409 (10)	0.0260 (3)	
H9A	0.1567	0.2942	0.0805	0.031*	
H9B	0.1739	0.3991	0.0862	0.031*	
C10	0.25342 (16)	0.34178 (9)	0.22886 (10)	0.0263 (3)	
H10A	0.2253	0.2876	0.2555	0.032*	
H10B	0.3746	0.3381	0.2427	0.032*	
C11	0.22973 (15)	0.42044 (8)	0.28392 (10)	0.0225 (2)	
C12	0.05790 (14)	0.45987 (8)	0.24136 (9)	0.0205 (2)	
C13	0.02759 (14)	0.53739 (8)	0.29541 (9)	0.0199 (2)	
H13A	0.0892	0.5268	0.3708	0.024*	
C14	0.09637 (14)	0.62177 (8)	0.27121 (9)	0.0200 (2)	
C15	0.01165 (15)	0.66138 (9)	0.17626 (10)	0.0229 (3)	
H15A	−0.0931	0.6372	0.1269	0.028*	
C16	0.07768 (15)	0.73634 (8)	0.15190 (9)	0.0225 (3)	
H16A	0.0175	0.7631	0.0867	0.027*	
C17	0.23114 (15)	0.77157 (8)	0.22307 (9)	0.0213 (2)	
C18	0.31465 (14)	0.73401 (8)	0.32119 (9)	0.0206 (2)	
C19	0.24887 (14)	0.65911 (8)	0.34429 (9)	0.0210 (2)	
H19A	0.3075	0.6330	0.4100	0.025*	
C20	0.54591 (18)	0.74117 (11)	0.48942 (10)	0.0346 (3)	
H20A	0.6472	0.7767	0.5304	0.052*	
H20B	0.5804	0.6802	0.4879	0.052*	
H20C	0.4668	0.7435	0.5198	0.052*	
C21	0.23844 (18)	0.87415 (10)	0.10281 (10)	0.0292 (3)	
H21A	0.2185	0.8253	0.0552	0.044*	
H21B	0.3167	0.9165	0.0959	0.044*	
H21C	0.1296	0.9031	0.0871	0.044*	

### Atomic displacement parameters (Å^2^)

**Table:**

	*U*^11^	*U*^22^	*U*^33^	*U*^12^	*U*^13^	*U*^23^
O1	0.0120 (4)	0.0367 (5)	0.0227 (4)	0.0006 (4)	0.0037 (3)	−0.0034 (4)
O2	0.0227 (5)	0.0359 (5)	0.0304 (5)	0.0013 (4)	0.0094 (4)	−0.0061 (4)
O3	0.0163 (4)	0.0326 (5)	0.0286 (5)	0.0025 (4)	0.0011 (4)	−0.0044 (4)
O4	0.0158 (4)	0.0308 (5)	0.0219 (4)	−0.0054 (4)	0.0021 (3)	−0.0042 (4)
O5	0.0195 (4)	0.0279 (5)	0.0243 (4)	−0.0053 (4)	0.0060 (4)	0.0001 (4)
C1	0.0141 (5)	0.0245 (6)	0.0237 (6)	0.0015 (4)	0.0072 (4)	0.0024 (5)
C2	0.0185 (5)	0.0238 (6)	0.0290 (6)	0.0017 (5)	0.0102 (5)	0.0021 (5)
C3	0.0197 (6)	0.0320 (7)	0.0388 (7)	0.0028 (5)	0.0148 (6)	−0.0020 (6)
C4	0.0181 (6)	0.0386 (8)	0.0367 (7)	0.0058 (5)	0.0108 (5)	0.0064 (6)
C5	0.0129 (5)	0.0376 (7)	0.0253 (6)	0.0017 (5)	0.0044 (5)	0.0022 (5)
C6	0.0139 (5)	0.0278 (6)	0.0230 (6)	0.0015 (5)	0.0071 (4)	0.0023 (5)
C7	0.0129 (5)	0.0268 (6)	0.0240 (6)	−0.0001 (4)	0.0065 (4)	0.0013 (5)
C8	0.0187 (6)	0.0313 (7)	0.0256 (6)	−0.0031 (5)	0.0082 (5)	−0.0054 (5)
C9	0.0188 (6)	0.0306 (7)	0.0284 (6)	−0.0010 (5)	0.0106 (5)	−0.0045 (5)
C10	0.0181 (6)	0.0266 (6)	0.0308 (7)	0.0027 (5)	0.0081 (5)	−0.0016 (5)
C11	0.0151 (5)	0.0238 (6)	0.0254 (6)	−0.0002 (4)	0.0065 (5)	0.0014 (5)
C12	0.0138 (5)	0.0229 (6)	0.0222 (5)	0.0005 (4)	0.0059 (4)	0.0007 (4)
C13	0.0121 (5)	0.0243 (6)	0.0199 (5)	0.0004 (4)	0.0043 (4)	0.0000 (4)
C14	0.0125 (5)	0.0241 (6)	0.0217 (5)	0.0013 (4)	0.0063 (4)	−0.0020 (4)
C15	0.0126 (5)	0.0263 (6)	0.0233 (6)	−0.0005 (4)	0.0023 (4)	−0.0013 (5)
C16	0.0159 (5)	0.0261 (6)	0.0204 (5)	0.0017 (5)	0.0039 (4)	0.0007 (5)
C17	0.0161 (5)	0.0227 (6)	0.0240 (6)	−0.0001 (4)	0.0081 (5)	−0.0030 (5)
C18	0.0112 (5)	0.0256 (6)	0.0214 (5)	−0.0005 (4)	0.0044 (4)	−0.0055 (5)
C19	0.0136 (5)	0.0270 (6)	0.0191 (5)	0.0018 (4)	0.0045 (4)	−0.0023 (4)
C20	0.0244 (7)	0.0513 (9)	0.0197 (6)	−0.0129 (6)	0.0027 (5)	−0.0044 (6)
C21	0.0289 (7)	0.0315 (7)	0.0244 (6)	−0.0070 (6)	0.0095 (5)	−0.0010 (5)

### Geometric parameters (Å, °)

**Table:**

O1—C6	1.3800 (15)	C9—C10	1.5205 (19)
O1—C7	1.3836 (14)	C9—H9A	0.9900
O2—C2	1.2231 (16)	C9—H9B	0.9900
O3—C11	1.2283 (15)	C10—C11	1.5103 (18)
O4—C18	1.3738 (14)	C10—H10A	0.9900
O4—C20	1.4297 (16)	C10—H10B	0.9900
O5—C17	1.3674 (15)	C11—C12	1.4736 (16)
O5—C21	1.4309 (16)	C12—C13	1.5108 (17)
C1—C6	1.3411 (17)	C13—C14	1.5277 (17)
C1—C2	1.4676 (17)	C13—H13A	1.0000
C1—C13	1.5099 (16)	C14—C15	1.3852 (17)
C2—C3	1.5122 (17)	C14—C19	1.4017 (16)
C3—C4	1.517 (2)	C15—C16	1.3975 (18)
C3—H3A	0.9900	C15—H15A	0.9500
C3—H3B	0.9900	C16—C17	1.3851 (16)
C4—C5	1.528 (2)	C16—H16A	0.9500
C4—H4A	0.9900	C17—C18	1.4074 (17)
C4—H4B	0.9900	C18—C19	1.3874 (18)
C5—C6	1.4935 (17)	C19—H19A	0.9500
C5—H5A	0.9900	C20—H20A	0.9800
C5—H5B	0.9900	C20—H20B	0.9800
C7—C12	1.3446 (17)	C20—H20C	0.9800
C7—C8	1.4906 (18)	C21—H21A	0.9800
C8—C9	1.5294 (17)	C21—H21B	0.9800
C8—H8A	0.9900	C21—H21C	0.9800
C8—H8B	0.9900		
			
C6—O1—C7	117.80 (9)	C9—C10—H10A	109.3
C18—O4—C20	116.55 (10)	C11—C10—H10B	109.3
C17—O5—C21	116.37 (10)	C9—C10—H10B	109.3
C6—C1—C2	119.46 (11)	H10A—C10—H10B	107.9
C6—C1—C13	122.55 (11)	O3—C11—C12	120.76 (12)
C2—C1—C13	117.98 (11)	O3—C11—C10	121.91 (11)
O2—C2—C1	120.73 (11)	C12—C11—C10	117.29 (11)
O2—C2—C3	120.81 (12)	C7—C12—C11	118.98 (11)
C1—C2—C3	118.43 (11)	C7—C12—C13	121.98 (10)
C2—C3—C4	113.83 (11)	C11—C12—C13	119.03 (10)
C2—C3—H3A	108.8	C1—C13—C12	108.70 (10)
C4—C3—H3A	108.8	C1—C13—C14	111.50 (10)
C2—C3—H3B	108.8	C12—C13—C14	110.69 (9)
C4—C3—H3B	108.8	C1—C13—H13A	108.6
H3A—C3—H3B	107.7	C12—C13—H13A	108.6
C3—C4—C5	111.70 (11)	C14—C13—H13A	108.6
C3—C4—H4A	109.3	C15—C14—C19	118.98 (11)
C5—C4—H4A	109.3	C15—C14—C13	120.72 (10)
C3—C4—H4B	109.3	C19—C14—C13	120.28 (11)
C5—C4—H4B	109.3	C14—C15—C16	121.10 (11)
H4A—C4—H4B	107.9	C14—C15—H15A	119.4
C6—C5—C4	110.85 (11)	C16—C15—H15A	119.4
C6—C5—H5A	109.5	C17—C16—C15	119.84 (11)
C4—C5—H5A	109.5	C17—C16—H16A	120.1
C6—C5—H5B	109.5	C15—C16—H16A	120.1
C4—C5—H5B	109.5	O5—C17—C16	124.70 (11)
H5A—C5—H5B	108.1	O5—C17—C18	115.81 (10)
C1—C6—O1	122.37 (10)	C16—C17—C18	119.48 (11)
C1—C6—C5	125.67 (12)	O4—C18—C19	124.51 (11)
O1—C6—C5	111.95 (11)	O4—C18—C17	115.33 (11)
C12—C7—O1	122.51 (11)	C19—C18—C17	120.13 (11)
C12—C7—C8	125.84 (11)	C18—C19—C14	120.35 (11)
O1—C7—C8	111.66 (10)	C18—C19—H19A	119.8
C7—C8—C9	110.54 (10)	C14—C19—H19A	119.8
C7—C8—H8A	109.5	O4—C20—H20A	109.5
C9—C8—H8A	109.5	O4—C20—H20B	109.5
C7—C8—H8B	109.5	H20A—C20—H20B	109.5
C9—C8—H8B	109.5	O4—C20—H20C	109.5
H8A—C8—H8B	108.1	H20A—C20—H20C	109.5
C10—C9—C8	109.91 (11)	H20B—C20—H20C	109.5
C10—C9—H9A	109.7	O5—C21—H21A	109.5
C8—C9—H9A	109.7	O5—C21—H21B	109.5
C10—C9—H9B	109.7	H21A—C21—H21B	109.5
C8—C9—H9B	109.7	O5—C21—H21C	109.5
H9A—C9—H9B	108.2	H21A—C21—H21C	109.5
C11—C10—C9	111.68 (11)	H21B—C21—H21C	109.5
C11—C10—H10A	109.3		
			
C6—C1—C2—O2	176.94 (12)	C10—C11—C12—C13	178.63 (11)
C13—C1—C2—O2	−1.98 (19)	C6—C1—C13—C12	−17.44 (16)
C6—C1—C2—C3	−1.40 (18)	C2—C1—C13—C12	161.44 (11)
C13—C1—C2—C3	179.68 (11)	C6—C1—C13—C14	104.85 (14)
O2—C2—C3—C4	156.87 (13)	C2—C1—C13—C14	−76.26 (14)
C1—C2—C3—C4	−24.78 (18)	C7—C12—C13—C1	19.86 (16)
C2—C3—C4—C5	49.18 (16)	C11—C12—C13—C1	−158.95 (11)
C3—C4—C5—C6	−47.29 (15)	C7—C12—C13—C14	−102.92 (13)
C2—C1—C6—O1	−176.73 (11)	C11—C12—C13—C14	78.27 (13)
C13—C1—C6—O1	2.14 (19)	C1—C13—C14—C15	−47.46 (15)
C2—C1—C6—C5	2.0 (2)	C12—C13—C14—C15	73.68 (13)
C13—C1—C6—C5	−179.18 (12)	C1—C13—C14—C19	134.08 (11)
C7—O1—C6—C1	13.08 (18)	C12—C13—C14—C19	−104.78 (12)
C7—O1—C6—C5	−165.77 (11)	C19—C14—C15—C16	1.38 (18)
C4—C5—C6—C1	23.06 (18)	C13—C14—C15—C16	−177.10 (11)
C4—C5—C6—O1	−158.14 (11)	C14—C15—C16—C17	0.62 (19)
C6—O1—C7—C12	−10.48 (18)	C21—O5—C17—C16	−5.67 (18)
C6—O1—C7—C8	169.57 (11)	C21—O5—C17—C18	173.51 (11)
C12—C7—C8—C9	−16.76 (18)	C15—C16—C17—O5	175.93 (11)
O1—C7—C8—C9	163.19 (11)	C15—C16—C17—C18	−3.22 (18)
C7—C8—C9—C10	48.72 (15)	C20—O4—C18—C19	−5.34 (18)
C8—C9—C10—C11	−57.64 (15)	C20—O4—C18—C17	176.71 (11)
C9—C10—C11—O3	−148.95 (12)	O5—C17—C18—O4	2.67 (16)
C9—C10—C11—C12	33.42 (16)	C16—C17—C18—O4	−178.10 (11)
O1—C7—C12—C11	171.57 (11)	O5—C17—C18—C19	−175.38 (10)
C8—C7—C12—C11	−8.48 (19)	C16—C17—C18—C19	3.84 (18)
O1—C7—C12—C13	−7.24 (19)	O4—C18—C19—C14	−179.71 (11)
C8—C7—C12—C13	172.71 (12)	C17—C18—C19—C14	−1.85 (18)
O3—C11—C12—C7	−177.87 (12)	C15—C14—C19—C18	−0.75 (17)
C10—C11—C12—C7	−0.21 (17)	C13—C14—C19—C18	177.73 (10)
O3—C11—C12—C13	0.98 (18)		

### Hydrogen-bond geometry (Å, °)

**Table:**

*D*—H···*A*	*D*—H	H···*A*	*D*···*A*	*D*—H···*A*
C3—H3A···O4^i^	0.99	2.41	3.3647 (18)	161
C5—H5A···O5^ii^	0.99	2.56	3.2366 (17)	126
C9—H9A···O2^ii^	0.99	2.55	3.3210 (17)	135
C10—H10B···O5^iii^	0.99	2.50	3.4822 (19)	173
C21—H21C···O2^iv^	0.98	2.57	3.1069 (18)	114

Symmetry codes: (i) *x*−1, *y*, *z*; (ii) −*x*, *y*−1/2, −*z*+1/2; (iii) −*x*+1, *y*−1/2, −*z*+1/2; (iv) *x*, −*y*+3/2, *z*−1/2.
